# Association analysis of DISC1 gene polymorphisms with Attention-Deficit Hyperactivity Disorder in Iranian population

**DOI:** 10.12669/pjms.315.8132

**Published:** 2015

**Authors:** Matin Kayyal, Abolfazl Movafagh, Mehrdad Hashemi, Arezou Sayad, Babak Emamalizadeh, Khashayar PourIran, Mohammadmoien Kayyal, Mohammad Reza Eslami Amirabadi, Mahdi Zamani, Hossein Darvish

**Affiliations:** 1 Matin Kayyal, Department of Molecular Genetics, Tehran Medical Branch, Islamic Azad University, Tehran, Iran; 2 Abolfazl Movafagh, Dept. of Medical Genetics, School of Medicine, Shahid Beheshti University of Medical Sciences, Tehran, Iran; 3 Mehrdad Hashemi, Department of Molecular Genetics, Tehran Medical Branch, Islamic Azad University, Tehran, Iran; 4 Arezou Sayad, Dept. of Medical Genetics, School of Medicine, Shahid Beheshti University of Medical Sciences, Tehran, Iran; 5 Babak Emamalizadeh, Dept. of Medical Genetics, School of Medicine, Shahid Beheshti University of Medical Sciences, Tehran, Iran; 6 Khashayar pourIran, Dept. of Medical Genetics, School of Medicine, Shahid Beheshti University of Medical Sciences, Tehran, Iran; 7 Mohammadmoien Kayyal, Department of Molecular Genetics, Tehran Medical Branch, Islamic Azad University, Tehran, Iran; 8 Mohammad Reza Eslami Amirabadi, Department of Psychiatry, Imam Hossein Medical Hospital, Shahid Behashti University of Medical Sciences, Tehran, Iran; 9 Mahdi Zamani Department of Medical Genetics, School of Medicine, Department of Neurology, Children’s Medical Center, Tehran University of Medical Sciences, Tehran, Iran; 10 Hossein Darvish, Dept. of Medical Genetics, School of Medicine, Shahid Beheshti University of Medical Sciences, Tehran, Iran

**Keywords:** Association, polymorphisms, ADHD, Hyperactivity, DISC1 gene, Iran

## Abstract

**Background & Objectives:**

Attention deficit hyperactivity disorder (ADHD) is a common heritable psychiatric disorder with a worldwide prevalence of 5%. The etiology of ADHD is still incompletely understood, but several studies, consistently indicate the strong role of genetic factors on this disorder. The aim of this study was to determine the effect of three SNPs rs11122319, rs11122330 and rs6675281 in the etiology of ADHD in an Iranian children

**Methods:**

In this research work, for the first time, we investigated the association of three SNPs (rs11122330, rs6675281 and rs11122319) in the *DISC1* gene with ADHD in Iranian population. Two hundred fourthy subjects composed of 120 patients and 120 healthy controls were included and tetra-primer ARMS PCR technique was used for genotyping all selected SNPs.

**Results:**

We found differences in genotype and allele distributions of rs 6675281 polymorphism between our patients and controls. The A, T and A alleles were the more frequent alleles in rs11122319, rs6675281 and rs11122330 polymorphisms in both case and control groups respectively. The TT genotype was more frequent in control group compared to patients. (*P* value = 0.008, OR= 1.5837, 95% CI= 1.1012 to 2.2776).

**Conclusion:**

Our findings strengthens the role of *DISC1* gene as a susceptibility locus for ADHD and indicate that rs6675281 polymorphism is a susceptibility factor for ADHD for the first time in children reported in an Iranian population in this part of the world.

## INTRODUCTION

Attention deficit hyperactivity disorder (ADHD) is a disorder affecting neurodevelopment, characterized by hyperactivity, inattention and impulsivity behavior. ADHD is a relatively common disorder with a prevalence rate of about 3-5% among prepubescent elementary school children.^1^,^2^ ADHD in childhood may show symptoms like learning disability, dysfunction in social actions, and increased risk for substance abuse.^3^,^4^

The high prevalence of Attention deficit hyperactivity disorder necessitates the understanding of the etiology, as well as the development of approaches for diagnosis and treatment of this disorder.^5^

However the etiology of ADHD is not clearly understood, it’s a highly heritable disorder of childhood^6^ and a complex disorder with additional moderate effects of multiple genes. Twin and adoption studies, have revealed that genetic factors play important roles in the etiology of ADHD. Significant associations have been shown between *DAT-1*, *DRD4*, *DRD5*, *5HTT*, *HTR1B* and *SNAP-25* genes and the ADHD.^5^,^7^

Disrupted-in-schizophrenia1 (*DISC1*) is a candidate gene causing susceptibility in a spectrum of psychiatric disorders.^8^ *DISC1* has the highest expression of brain tissue in the hippocampus and the cerebral cortex^9^ and have been implicated to be involved in neuronal migration, neurite outgrowth and axon targeting during brain development.^10^ *DISC1* is located on chromosome 1q42, and originally identified in a Scottish pedigree by observing a breakpoint causing a chromosomal translocation. Spanning over 410 kb it includes 13 exons and encodes a cytosolic scaffold protein which acts in collaboration with several proteins with different functions.^10^ Being one of the most promising candidate genes for major neurodevelopmental disorders, *DISC1* have been shown to be associated with some mental disorders such as depression, bipolar disorder, schizophrenia, and schizoaffective disorder in several independent populations and according to its function, *DISC1* may contribute to susceptibility to psychiatric disorders.^10^ The symptoms of Attention-deficit/hyperactivity disorder (ADHD) are similar to those of BPD in several aspects, so it’s very promising to find some relations between ADHD and *DISC1* gene. In one study performed in 2013 it was shown that disc1 variants have associations with ADHD in Norwegian and Spanish patients.^11^

In this study we selected rs11122319, rs11122330 and rs6675281 polymorphisms all previously shown to be associated with several mental illnesses,^11^-^13^ for investigation. The aim of this study was to determine the effect of three SNPs rs11122319, rs11122330 and rs6675281 in the etiology of ADHD in an Iranian children.

## METHODS

### Subjects

The study was performed on a total of 240 peripheral blood samples consisted of 120 unrelated ADHD patients at based referral and teaching hospital affiliated to Shahid Beheshti University of Medical Sciences and 120 healthy controls without any history of mental or neurodegenerative illness in their family. All patients were selected by neurologists according to DSM IV criteria, all were of combined type and just sporadic cases were included in the study. All of the patients were between ages 7 and 12 and consisted of 61% males and 39% females. The patient and control groups were similar in terms of mean age and gender distributions and both were of Iranian origin. Study and patients were assigned on the basis of national/international behavior hereditary and neurologists according to DSM IV criteria, protocols and approved according to local law and regulations, by the Institutional Review Boards of each participating referral hospital.

### DNA isolation and SNP genotyping

DNA isolation was performed on peripheral blood using the standard salting out method. SNPs were genotyped by Tetra-Primer ARMS PCR technique. The PCR was performed with personal thermal cycler (Techne, Genius, UK) with 50 µl of reaction volume and final primer concentration of 0.2 mM using the Ampliqon® master mix (1.5 mM MgCl2) and the primers shown in Table-I. The PCR reaction consisted of 30 cycles at 95°^C^ for 40 s, 57.2°^C^ for 30s, and 72°^C^ for 40s. The PCR products were electrophoresed on 2% agarose gel to observe the bands illustrated in Table-I. The results were confirmed by random sequencing of 50 samples.

**Table-I:** PCR primers and PCR product sizes.

SNP	Primers	Band Sizes
rs11122330	FI: GAGATGCTCAGTGGTCATTTATCAAGTAAA	A allele: 146
	RI: TATGGAGCGAACTATGGGGAACACAC	G allele: 193
	FO: TTGGACATGAGTACAGTGTTCATTTTCC	Outer primers: 283
	RO: GTCACCGCACTTACCTAATCTCTCTGAA	
rs6675281	FI: AAACCATTTCTGGACGGCTAAAGCCT	A allele: 205
	RI: TGATGTTAATGATCTAATCTCCTCGGTTAG	G allele: 258
	FO: AAGACGGTGATTTTTCCAGTTTAAGGAG	Outer primers: 407
	RO: GTTGTGCACTACCTACCATAGGCAGTCT	
rs11122319	FI: CCTGGTTGACTTTCTTGTCCTGCTACA	A allele: 196
	RI: TGTGCTTCAAACAAACACACAAACACAC	G allele: 270
	FO: GGACATGAACTCATTTGAAATGGCTAGG	Outer primers: 411
	RO: AGGACATGTACAAAGATCCACAGAAGGG

FI: Forward Inner primer; RI: Reverse Inner primer; FO: Forward Outer primer; RO: Reverse Outer primer.

### Statistical analysis

Fisher exact test was used to compare genotype and allele frequencies between ADHD cases and controls. Odds ratio (OR) together with 95% confidence interval (CI) was estimated; and a *p* value less than 0.05 was considered as statistically significant for the tests. We used Statistical Software Package for the Social Science (SPSS 18.0, Chicago) to perform statistical analysis.

## RESULTS

Three single nucleotide polymorphisms, rs11122319, rs11122330 and rs6675281 were examined in this study for genotype and allele frequencies in patient and control groups. The polymorphisms were investigated in 120 ADHD patients and 120 healthy controls with mean age of 9.5 ± 2.5 and 9 ± 2.9 respectively. There was no significant differences in age and gender distributions between two groups and the genotype distributions of all three polymorphisms did not deviate from Hardy-Weinberg equilibrium expectation.

The allele and genotype frequencies are shown in Table-II. The A, T and A alleles were the more frequent alleles in rs11122319, rs6675281 and rs11122330 polymorphisms in both case and control groups respectively. One of the SNPs, rs6675281, showed marginal association of 0.005 (OR= 0.4080, 95% CI = 0.2314 to 0.7193), so that the TT genotype was more frequent in control group compared to patients. The allelic association was also observed for this polymorphism (*P* value = 0.008, OR= 1.5837, 95% CI= 1.1012 to 2.2776). For other two polymorphisms, no significant differences in genotypic and allelic distributions were shown. There was no linkage disequilibrium between three SNPs. *p* value cut-off to consider each as significant is 0.016 (Bonferroni correction). Hence rs6675281 with p values 0.008 and 0.005 remained to be significant (Table-II).

**Table-II:** Frequencies of rs11122319, rs6675281, rs11122330 polymorphisms.

Frequencies of rs11122319 polymorphism			
Allele frequencies	ADHD (%)	Control (%)	p value
A	122 (50.8)	117 (48.75)	0.35
G	118 (49.2)	123 (51.25)	
Total	240 (100)	240 (100)	

Genotype frequencies			
AA	29 (24.2)	31 (25.8)	0.44
AG	64 (53.3)	55 (45.8)	
GG	27 (22.5)	34 (28.4)	
Total	120 (100)	120 (100)	

Frequencies of rs6675281 polymorphism			
Allele frequencies	ADHD (%)	Control (%)	p value
T	122 (50.8)	149 (62)	0.008
C	118 (49.2)	91 (38)	
Total	240 (100)	240 (100)	

Genotype frequencies			
TT	28 (23.4)	47 (39.2)	0.005
TC	66 (55)	55 (45.8)	
CC	26 (21.6)	18 (15)	
Total	120 (100)	120 (100)	

Frequencies of rs11122330 polymorphism			
Allele frequencies	ADHD (%)	Control (%)	p value
A	139 (57.9)	132 (0.55)	0.29
G	101 (42.1)	108 (0.45)	
Total	240 (100)	240 (100)	

Genotype frequencies			
AA	39 (32.5)	34 (28.3)	0.28
AG	61 (50.8)	64 (53.3)	
GG	20 (16.7)	22 (18.4)	
Total	120 (100)	120 (100)	

## DISCUSION

DISC1 was discovered in a Scottish pedigree in which a chromosomal translocation that breaks this gene segregates with psychiatric disorders, mainly depression and schizophrenia. Linkage and association studies in diverse populations support DISC1 a susceptibility gene to a variety of neuropsychiatric disorders. (DISC1) gene, located in a 415 kb region on chromosome 1q^42.1^. DISC1 has since been implicated in several psychiatric disorders, including autism spectrum disorders, and cognitive functions such as sustained attention and visual working memory.^11^

One hundred twenty children with ADHD and 120 healthy controls were selected for each of three polymorphisms and result revealed that the rs6675281 polymorphism had marginal association with ADHD in our sample population of children. The TT genotype of the mentioned SNP showed differences in distribution in patient and control groups and were more frequent in healthy controls. Hence, it have negative association with ADHD. In the analysis of allelic frequencies, the results showed allelic association for this polymorphism as well. Therefore, there was no other significant differences in genotypic and allelic frequencies for other polymorphisms between two groups.

In the first and only existing study regarding the effect of *DISC1* variants on ADHD, Jacobsen and colleagues reported the association of rs6675281 and rs11122330 polymorphisms with ADHD in 694 ADHD cases and 735 normal controls in Norwegian population.^11^ The detail In the ADHD case/control analysis by Jacobsen KK and coworker in 3013 found an association for the intronic DISC1 SNP, rs1538979 (OR: 1.33, 95% CI 1.03–1.73, P¼0.03), which was further strengthened using a Spanish cohort for replication (meta-analysis OR 1.25, P¼0.008 for the tested tag-SNP rs11122330). The rs11122330/rs1538979 markers have been studied by several different groups.^11^ Hennah et al. [2009] found different trends in the different cohorts for SCZ and BPD, both regarding risk allele and gender specific associations. In contrast to our results, they also reported an interaction between rs1538979 and rs821633. This could indicate that the true risk locus. resides elsewhere on a haplotype marked by these two SNPs, or alternatively, that there are several risk variants in DISC1, or that the signal is secondary to another gene in the region.^12^,^13^

In other previous studies performed on *DISC1* polymorphisms, the rs11122319 polymorphism was showed to be associated with mean temporal cortical thickness in patients with history of psychosis by.^14^ The rs6675281 single nucleotide polymorphism had inconsistent results about association with ADHD in several association studies in different populations. It was reported to be associated with schizophrenia in French and Algerian patients and the C allele was over-transmitted in patients.^15^ This polymorphism was also associated with striatal and hemisphere volumes^16^ and gray matter volumes,^17^ all known to be risk factors for mental disorders and specifically schizophrenia. In some other studies, no association between the rs6675281 polymorphism and ADHD was observed.^18^-^21^ Finally in one investigation on the effects of *DISC1* variants, it was implicated that the rs6675281 polymorphism’s alleles affect the expression level of *DISC1*, so that the T allele carriers had significantly higher levels of *DISC1* expression in comparison with C allele carriers.^22^,^23^

According to the overall reports, our results of rs6675281 single nucleotide polymorphism was in consistence with several other studies, and given the association of T allele of this SNP with higher expression of *DISC1*, more frequency of T allele in our normal samples seems to be logical. Our results replicated the involvement of *DISC1* and its variants in the etiology of ADHD. According to limited number of our ADHD samples due to limited accessibility to valid cases. to our knowledge, our study is the first research investigation of DISC1 in ADHD and it adds ADHD to the traits possibly associated with DISC1 variation in children in Iran and Middle East. Although DISC1 for a long time was considered a susceptibility gene for psychotic disorders, more recent findings have shown that it is involved in general neurodevelopment and signaling, and it is possible that unknown functional variants may predispose an individual for a range of different mental illnesses. More functional genomics studies could be cited and discussed with review article entitle DISC1 mouse models as a tool to decipher gene-environment interactions in psychiatric disorders.^24^,^25^

This research work needs much more data in different populations and ethnic groups to further support of the role of DISC1 in development of ADHD and draw firm conclusion.
